# Decision-making psychology and method under zero-knowledge context

**DOI:** 10.1038/s41598-022-06753-z

**Published:** 2022-02-24

**Authors:** Neng-gang Xie, Meng Wang, Ya-yun Dai, Ye Ye, Joel Weijia Lai, Lu Wang, Kang Hao Cheong

**Affiliations:** 1 School of Management Science and Engineering, Anhui University of Technology, Ma’anshan, 243002 Anhui China; 2 School of Business, Anhui University of Technology, Ma’anshan, 243002 Anhui China; 3 School of Mechanical Engineering, Anhui University of Technology, Ma’anshan, 243002 Anhui China; 4 Science, Mathematics and Technology Cluster, Singapore University of Technology and Design (SUTD), 8 Somapah Road, Singapore, 487372 Singapore

**Keywords:** Biological techniques, Behavioural methods, Psychophysics

## Abstract

For a certain kind of decision event, the decision maker does not know the internal mechanism and knowledge information of the decision events.When this kind of decision events gives multiple selection branches, it is found that there is a decision psychological tendency to find the most common features by comparing the selection branches. Based on this, a zero-knowledge decision making (ZKDM) method is proposed. By defining the feature points and feature sets of the selection branches of the decision events, the characteristic moments of the system are constructed and the branch with the most common characteristics is obtained. It is observed that through the findings of investigation the probability of arriving at the correct choice based on the ZKDM method is high. The effectiveness of the ZKDM method may be related to the fact that the designers of decision events usually determine the correct selection branch first, before changing it to design other branches. A questionnaire survey of 279 respondents reveals that more than half of them actually adopt such a design idea. Furthermore, a separate questionnaire survey of 465 decision-makers reveal that 19.14% of the respondents clearly adopt ZKDM.

## Introduction

Decision making is often a black-box, but remains a key feature in many real-world scenarios. Decision making often involve mechanisms and causation to choose the best action available among the presented options. Human decision making can be modelled from the computational perspective. This is often dependent on implied theories and resource constraints. Traditionally, algorithms are believed to be the saving grace to the limitations of human judgement in decision making^1,2^. For example, computational complexity theory provides a way for modelling and quantifying human decision making as a function of computational complexity^3^. Other methods include the entropy weight method which places weights on certain options to accurately reflect the amount of information provided by each option, while limiting the interference of human factors. This is advantageous as it presents the decision event independently from the characteristics of the decision maker^4^. Since the advent of computers, algorithmic decision-making models have taken the forefront in research to examine factors that influence individual and organisational decisions^5,6^.

The nature of the decision events widely demands different decision-making algorithms. In particular, for single-agent selection problems, there is a need to computationally represent each option, place weight on priorities, and consistency in measuring options; these can be extended to multi-criteria decision making, commonly known as Analytic Hierarchy Process^7^, and often involve fuzzy set theory. These often require comprehensive fore-knowledge of the decision space, which is formed by the overlap of multiple decision variables, leading to the emerging use of fuzzy logic in multi criteria problems^8–10^. Often, decision-makers assign variable weights to synthesise measures to risks on certain options, especially in multi-attribute decision making scenarios^11^.

The above decision-making algorithms belong to the category of rational decision-making. Decision makers need to rely on professional (or specialized) knowledge such as information, rules, theories, methods, and mechanisms related to the decision-making event itself. In special cases of decision events, it is possible for a decision maker to arrive at a choice without having fore-knowledge of the decision events. To solve this kind of problems, decision makers mostly rely on intuition for decision-making, such as probabilistic speculation, fuzzy impression, experience transfer of similar problems, psychological tendency, etc. Especially, one of the tendentious decision-making psychology deserves attention. Here, we will take a multiple-choice question as an example to illustrate this psychological tendency and decision method.

Consider the following example in the form of a multiple-choice question: Which is the actual Chinese social media and multipurpose application WeChat logo? Those images of the WeChat logo are referenced from question 3 of this link: https://www.uisdc.com/official-logo-design-quiz and the accessed date was on February 10, 2020 (if this link fails or expires, please visit this website:  https://osf.io/58cjp/?view_only=c9bc552dce63439b828bd09a0b491c2d).  Note that options A, B, C and D in the original images of question 3 are replaced by options 3, 4, 1 and 2 in our example, respectively.

There are three main ways in which decision-makers can arrive at the correct choice: (i) they remember (or have a former impression of) the WeChat logo; (ii) they choose at will and randomly chose the right option; or (iii) after careful observation of the options, they use a certain method of analysis to arrive at the correct choice. Here, the third type of decision-makers, employ the method of observation, thinking and selection is of interest in this paper. The third type of decision makers may adopt a decision-making psychology that tend to arrive at the the answer that has the most common characteristics with the other selection branches. The specific decision-making methods are as follows: by observing the four options of the WeChat logo, it is found that the discrimination of the four options is mainly depicted in the following three feature points: (i) the arrangement of the left and right positions of the speech bubbles; (ii) the dividing line between the big bubble and the small bubble; and (iii) the mouth arc on the small bubble (a smiling face). The feature set based on the first feature point is (the small bubble is on the left and the big one is on the right) in option 1, while in options 2, 3 and 4 (the small bubble is on the right and the big one is on the left) respectively. The feature set based on the second feature point is (there is a dividing line between the two bubbles) in options 1, 2 and 3, while in option 4 (there is no dividing line between the two bubbles). The feature set based on the third feature point is (the small bubble has no mouth arc) in in options 1, 3 and 4, while in option 2 (the small bubble has a mouth arc). Therefore, based on the similarities and differences of the above three feature points, option 3 has the most common characteristics and is unique, and the correct answer is indeed the third option.

This begs the following questions: (1) How universal are the cases like the above mentioned WeChat logo where the most common characteristics can be found in the selected branches? (2) What is the proportion of decision makers with this decision-making psychology and using this decision-making method in the population? (3) What is the mechanism behind this decision-making psychology? (4) Is this method correct? We provide answers to these questions in sections “Method” and “Results”.

## Method

### ZKDM method

To facilitate the research, firstly, the relevant concepts and definitions in “zero-knowledge proof” (the certifier can make the verifier believe that a certain conclusion is correct without providing any useful knowledge to the verifier) are used to define the “zero-knowledge decision”: For a certain kind of decision events, the decision-maker, without knowing the internal mechanism and knowledge information of the event, can make a correct decision inferred from the alternative branches provided by the event designer. In order to facilitate the standardization and quantification of theoretical analysis, the characteristics of selected branches are mathematically expressed, and the common characteristics are characterized by defining the index of system characteristic moment. A smaller system characteristic moment of selected branches will yield more common characteristics.

The zero-knowledge decision-making process is: suppose that a decision event *D* has *n* alternative branches. There are $m>1$ types of feature points to effectively distinguish *n* alternative branches; among which the selection of feature points includes words, numbers, graphics, attributes, symbols, locations, categories, etc. The feature set ${\mathbf {C}}_i=(C_{1i},C_{2i},\ldots ,C_{ni}),~i=1,2,\ldots ,m$ is defined as the representation of *n* selection branches at the *i*-th feature point. For the feature set ${\mathbf {C}}_i$, the characteristic moment of the selection branch *j* on the feature point *i* is defined as

1 $$\begin{aligned} L_{ji}=\sum _{k=1,~k\ne j}^n l_{jk},\quad j=1,2,\ldots ,n;~i=1,2,\ldots ,m, \end{aligned}$$

where

2 $$\begin{aligned} {\left\{ \begin{array}{ll} l_{jk}=0 &{}\quad \text {if } C_{ji}=C_{ki} \\ l_{jk}=1 &{}\quad \text {if } C_{ji}\ne C_{ki} \end{array}\right. }. \end{aligned}$$

The system characteristic moment of the selection branch *j* at all *m* types of feature points is

3 $$\begin{aligned} L_{j}=\sum _{i=1}^m L_{ji},\quad j=1,2,\ldots ,n. \end{aligned}$$

The corresponding unique branch given by $\min (L_1,L_2,\ldots ,L_n)$, is the selection result based on the ZKDM method.

The events that can make use of the ZKDM method need to satisfy the following three conditions:

1. The event designers need to provide a finite number of *n* options, including the correct one. Further, the branches are different from each other, that is, any two branches *j* and *k*, $j,k\in \{1,2,\ldots ,n\}, j\ne k$, there is at least one characteristic point *i*, satisfying $C_{ji}\ne C_{ki}$, $i\in \{1,2,\ldots ,m\}$.
2. The *m*, $m>1$, types of feature points can be set to effectively distinguish *n* selection branches provided by the event designer. *m* is necessarily greater than 1. If $m = 1$, in order to realize the effective differentiation of *n* selection branches on this unique feature point, the elements in the set ${\mathbf {C}}_1=(C_{11},C_{21},\ldots ,C_{n1})$ must be completely inconsistent (different from each other). Hence the system characteristic moment $L_1=L_2=\cdots =L_{n}=n-1$ of all selection branches leads to the non-uniqueness of the corresponding branches of $\min (L_1,L_2,\ldots ,L_n)$.
3. The elements in the feature set ${\mathbf {C}}_i$ for $i=1,2,\ldots ,m$ are not all consistent (there are at least two different elements) and not all inconsistent (there are at least two identical elements). 1) There are at least two different elements. If all the elements in the feature set are the same, then the feature point is not distinguished and cannot be a feature point. 2) There are at least two identical elements. If all elements in the feature set are different, then the characteristic moment of all alternative branches at the feature point are $n-1$. As a result, the characteristic moment at the feature point has no effective contribution to obtain the corresponding branches of $\min (L_1,L_2,\ldots ,L_n)$.

For the four options of the WeChat logo, the decision-making process using the ZKDM method is presented. For each feature point, a feature set can be established to describe the characteristics of the four pictorial options. The feature set based on the first feature point is { (the small bubble is on the left and the big one is on the right), (the small bubble is on the right and the big one is on the left), (the small bubble is on the right and the big one is on the left), (the small bubble is on the right and the big one is on the left)}. The feature set based on the second feature point is {(there is a dividing line between the two bubbles), (there is a dividing line between the two bubbles), (there is a dividing line between the two bubbles), (there is no dividing line between the two bubbles)}. The feature set based on the third feature point is {(the small bubble has no mouth arc), (the small bubble has a mouth arc), (the small bubble has no mouth arc), (the small bubble has no mouth arc)}. For the above feature sets, the characteristic moments of the four options of the WeChat logo for the three feature points are calculated respectively according to Equation (1). The characteristic moments of the four pictures corresponding to the first, second and third feature points are (3, 1, 1, 1), (1, 1, 1, 3), and (1, 3, 1, 1), respectively. By summing up the characteristic moments of the above three characteristic points, the characteristic moments of the system are (5, 5, 3, 5). The system characteristic moment of the third option is the smallest, so the decision-maker should choose option 3.

### Field investigation

Firstly, we examine the potential use of ZKDM through a questionnaire, which has been approved by the academic ethics committee of Anhui University of Technology. We confirm that all methods were carried out in accordance with relevant guidelines and regulations and informed consent was obtained from all subjects. Through a series of examination papers, we notice that ZKDM can be used in some cases. See Appendix for a compilation of such cases. Findings from our investigation, in Appendix, also reveal that ZKDM can be adopted in events of multi-disciplinary decision making, for instance, mathematics, physics, chemistry, geography, history, biology and language. Thus, this method has certain universality. At the same time, cases with ZKDM also appear in the representative decision making situations like the Chinese college entrance examination. This case indicates that the designers of decision-making events have not yet realized (or ignored) the existence of ZKDM.

We conducted multiple surveys on decision-makers to elucidate the real-world extent of the use of the ZKDM method. We designed two questionnaires. The first questionnaire consists a single question where respondents were instructed to answer the multiple-choice question in “Introduction”, and the second questionnaire was to extract the reason for their choice. Three options were given in the questionnaire as possible reasons: (i) I remember the real WeChat logo. (ii) I choose at random and it turns out to be correct. (iii) After careful observation and thinking, I used a certain decision making approach. If respondents choose the third option, they were requested to explain the ideas or methods used to arrive at their choice.

The respondents (freshmen of Business School of Anhui University of Technology) were divided into three groups. The first group was told that the questionnaire had no purpose, the second group was told that the questionnaire was related to the abilities of evaluation, analysis and reasoning, and the third group was told that the questionnaire was related to the selection of innovation tournament. The questionnaires of the three groups were carried out simultaneously. Questionnaire 1 was conducted first. Then, the organizers checked the answers of each student, and the ones who selected the correct option stayed to participate in Questionnaire 2. The processes relating to the conduct of the questionnaires were performed in strict accordance with the requirements of the examination discipline.

## Results

**Figure 1:**
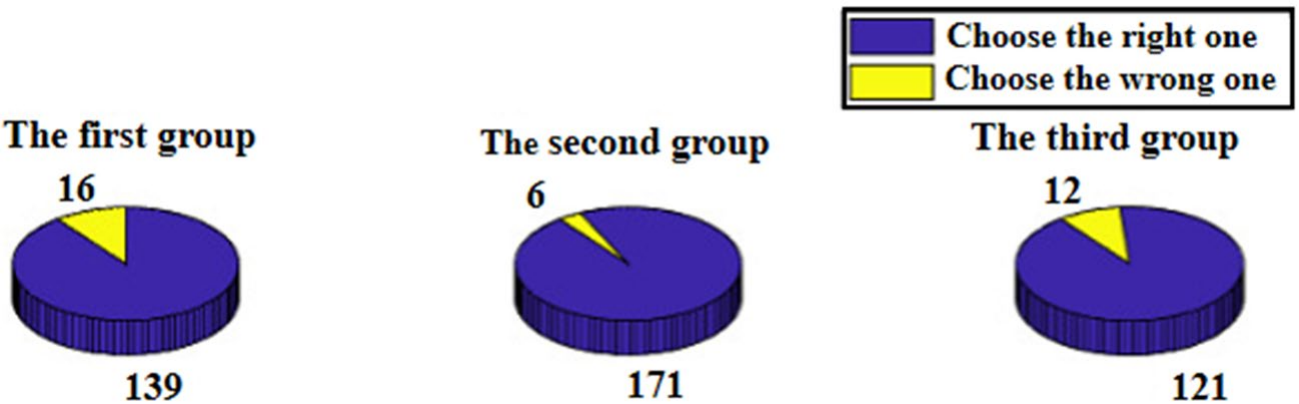
Statistics of the number of decision-makers, and the results in Questionnaire 1.

**Figure 2:**
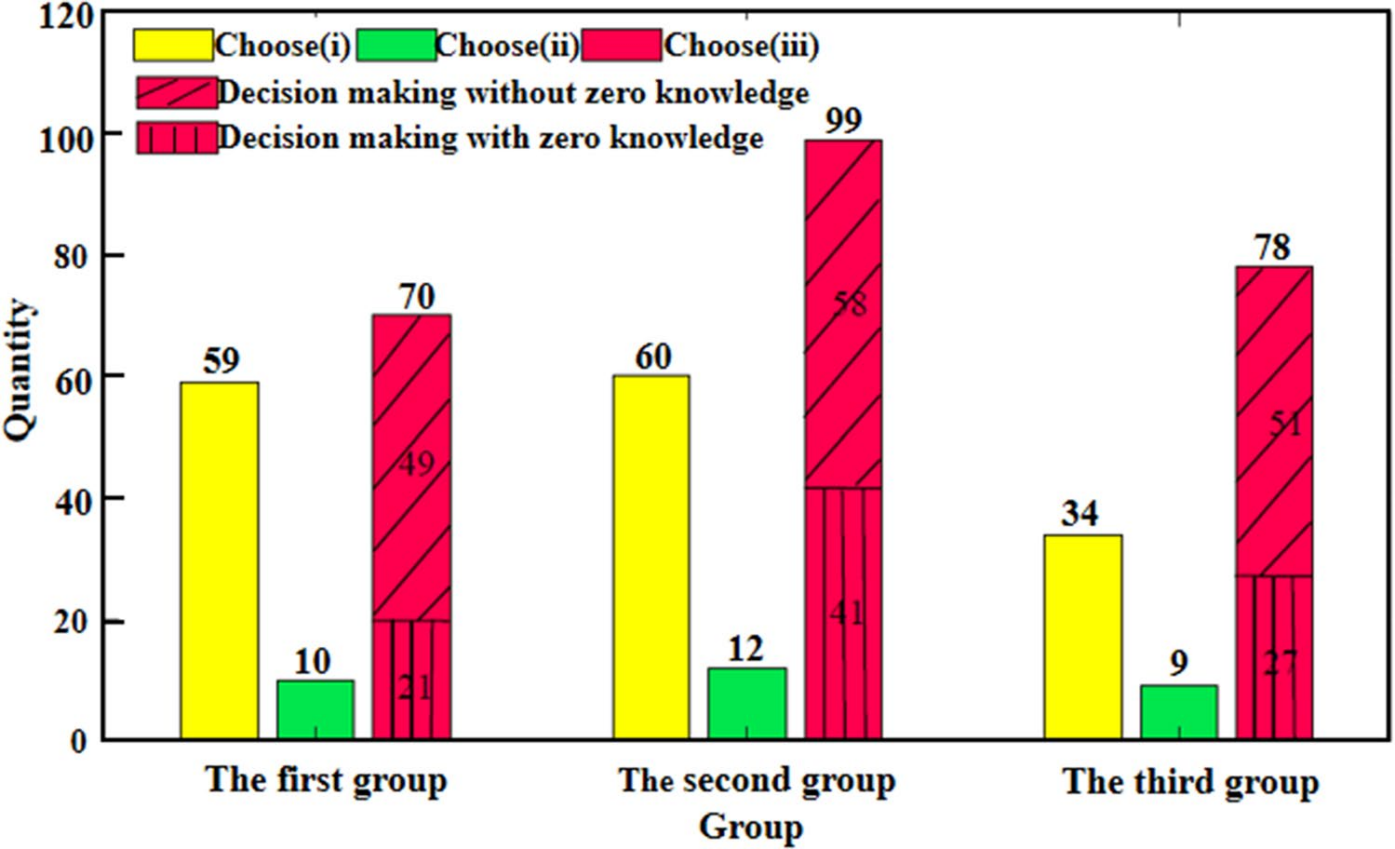
Statistics of the number of decision-makers in Questionnaire 2.

Figures 1 and 2 present the findings of investigation of Questionnaire 1 and Questionnaire 2, respectively. In response to Questionnaire 2, some keywords appear, for instance, “having similarity”, “having the maximum common features”, “combining the most features” and “difference exclusion”. The organizers screened and judged them, and divided them into two categories: irrelevant and relevant to ZKDM.

According to the findings of investigation of Questionnaire 1, we observed that the proportions of respondents who chose the correct choice in all three groups are high, 89.68%, 96.61% and 90.98%, respectively. The reason may be that WeChat is widely and frequently used, and the respondents have familiarity with the correct logo. The reason why the proportions in the second and third groups are higher than the one in the first group may be that the respondents were informed of the purpose of the questionnaire in advance. It aroused the respondents’ attention to the questions in the questionnaire.

To elucidate the reasons for each correct response, a second questionnaire was conducted. The findings from investigating the responses from Questionnaire 2 reveal that the proportion of the respondents with option (iii) was 50.36%, 57.89% and 64.46% of all respondents (that is, those who answered Questionnaire 1 correctly) in each group, respectively. The proportion respondents whose decision making is related to ZKDM in each group accounted for 15.11%, 23.98% and 22.31% among all the respondents in Questionnaire 2; 13.55%, 23.16% and 20.30% among all the respondents in each group; and 30.00%, 41.41% and 34.62% among all the respondents with option (iii) in each group, respectively.

In the second and third groups, the proportions of respondents with option (iii) and those related to ZKDM are relatively high. The reason may be related to informing the purpose of the questionnaire in advance. The purposes of the abilities of “evaluation, analysis and reasoning” and the selection of “innovation tournament” have played a certain motivating role for the respondents. In the third group, the proportion of respondents selecting option (iii) was 64.46%, higher than that of the second group, 57.89%. However, the proportion of the respondents indicating hints of using ZKDM in the third group was 34.62%, lower in comparison to 41.41% in the second group. The reason may be related to the different purposes informed to the two groups of questionnaires. The purpose of the questionnaire of abilities of “evaluation, analysis and reasoning” may guide the respondents to make more rational reasoning and thinking, so the proportion of the responses related to ZKDM in the second group is relatively higher. The purpose of the questionnaire as a selection for an “innovation tournament” may guide the respondents to think more creatively to showcase innovation abilities. In the responses from the third group, there are descriptions related to design layout, meaning behind the logo and aesthetics. Some examples of responses given by various respondent in the third group are: “the layout of big bubble on the left and small bubble on the right is pleasing to the eyes”, “it looks better with shadow on the edge”, “according to the aesthetics of the picture, the large chat icon should be in the back and the small one in the front and it is more beautiful to look from left to right in accordance with the reading style”, “WeChat has no meaning of smiling”, “WeChat app means to provide a platform for people to people to communicate, but at the same time, it will protect people’s privacy. It has a sense of boundary but not complete integration”, “we can get closer to each other through the news from time to time, but there is still a distance and will not merge”, and “the person that initiates the message has a stronger desire to communicate and thus has a bigger speech bubble”. These diverse thoughts were reasons provided by respondents in the third group for choosing option (iii). However, we also caution that there may be speculators which may skew our analysis, that is, the respondents who choose the right WeChat logo because of their memory or good luck, or for utilitarian goal (expecting to be selected to participate in the innovation tournament), they chose option (iii) but wrote far-fetched ideas and methods (independent of ZKDM) that embody the aspect of innovation.

Next, we explain the mechanism behind ZKDM, and why it works. The mechanism behind the ZKDM method may be attributed to the subjective design idea of the designer of the decision event. The designer of the decision event (multiple-choice questions in our case) usually first determines the correct option, and then make perturbing changes to the correct option so as to design other alternative branches. Methods of making changes mainly include the principle of similarity (or difference) in shape and the principle of proximity (or opposition) in meaning, and this change may only be presented in a feature point. Therefore, according to this idea the core of all the selection branches is the correct one, which must be the branch with the most common characteristics with other branches. The ZKDM method is just the inverse process of the above design idea, so the quantitative method can be used to establish the system characteristic moment and the selection branch with most common features can be obtained.

Then does the designer who uses the above design ideas to design decision events (multiple-choice questions) exist? How broad and universal is it? For this reason, we designed a questionnaire for question designers. It contains two questions: (i) When you design the selection branches of multiple-choice questions, do you first determine the correct answer? A. Yes; B. No (please write down your specific method). (ii) How do you design and determine the other choices except the correct answer? A. Subjective imagination and determination at will; B. Change the correct answer to generate other options; C. According to the wrong ideas easily induced by the investigated knowledge points, the remaining selection branches are designed; D. According to the specific situation of the exercise problems, the above methods (please check: A, B, C) have all been used; E. Use other methods to design and determine (please write down your method).

The results of the survey by questionnaire of 279 teachers in Anhui University of technology showed that for the first question, 274 candidates chose A and 5 candidates chose B. This result indicated that the vast majority (98.21%) of designers first decide the right choice. For the second question, 11 candidates chose A, 44 candidates chose B, 87 candidates chose C, in option D with multiple choices: 1 candidate chose A and B, 3 candidates chose A and C, 67 candidates chose B and C, 66 candidates chose A, B and C, and no one chose option E. The proportion of the number of choosing option B individually in the total number is 15.77%. The proportion of the number of candidates (178 candidates) choosing options that include option B individually and option D with option B accounted for 63.80% of the total number of candidates. The number of choosing options that includes option B (178 times) accounted for 36.93% of the total number of options (482 times). Therefore, it is common for designers to “change the correct answer to derive the remaining options”. Hence, we can extrapolate that this design method to craft selection branches for decision events can be considered universal.

Finally, we prove the correctness of the ZKDM method. Assume that the designer of the decision event designs the interference branches by making changes to the characteristic points of the correct branch, and the changes of each interference branch on the same characteristic point are different, we can prove that the system characteristic moment of the correct branch is the smallest and unique.

It is assumed that when the designer of the decision event designs the *j*-th interference branch, a number of $h_{j}$, $1\le h_{j}\le m$, feature points of the correct branch is subjected to change. However, for the change of the same feature point, different interference branches must be different. A number of $N_i$ interference branches is designed based on the change of the *i*-th, $(i=1,2,\ldots ,m)$, feature point. Since there are at least two different elements in the feature set corresponding to any feature point, $N_{i}\ge 1$. Furthermore, there are at least two identical elements at the same time, thus $N_{i}<n-1$. According to the assumption, for the same feature point *i*, the elements in the feature set ${\mathbf {C}}_i=(C_{1i},C_{2i},\ldots ,C_{ni})$ corresponding to the interference branches are not only different from the elements of the correct branch, but also different from each other. The set of serial numbers corresponding to the number of $N_i$ interference branches is recorded as ${\mathbf {S}}_i$. There exists

4 $$\begin{aligned} {\mathbf {S}}_1\cup {\mathbf {S}}_2\cup \dots \cup {\mathbf {S}}_m=\bar{{\mathbf {S}}}, \end{aligned}$$

where $\bar{{\mathbf {S}}}$ is the set composed of the sequence numbers of all interference branches.

For the feature set ${\mathbf {C}}_i$, the feature moment of the branch with the same elements as the correct branch (including the correct branch) is $N_i$. For the branches with different elements from the correct branch, because they are also different from each other, the feature distance is $n-1$. The system characteristic moment of the correct branch on all *m* characteristic points is $\sum _{i=1}^m N_{i}$. For an arbitrary interference branch $j\in {\mathbf {S}}_x (x\in \{ 1,2,\ldots ,m\})$, ${\mathbf {S}}_x$ represents a set containing the serial numbers of the *j*-th interference branch (in all, there are $h_j$ sets of this type), and its system characteristic moment on all *m* characteristic points is $\sum _{i=1,i \ne x}^m N_{i}+h_{j}(n-1)$. Since

5 $$\begin{aligned} N_{i}<n-1\quad (i=1,2,\ldots ,m), \end{aligned}$$

then

6 $$\begin{aligned} \sum _{i=1}^m N_{i}=\sum _{i=1,i\ne x}^m N_{i}+\sum _{i=1,i=x}^m N_{i}<\sum _{i=1,i \ne x}^m N_{i}+h_{j}(n-1). \end{aligned}$$

Thus, the system characteristic moment of the correct branch is unique and the smallest.

The interference branch is designed by assuming that the designer of the decision event only selects to change a feature point of the correct branch. This situation is a special case under said circumstances, that is, $h_{j}=1$ for all interfering branches. In this instance, $\sum _{i=1}^m N_{i}=n-1$ and ${\mathbf {S}}_a\cap {\mathbf {S}}_b=\Phi ~(a,b\in \{1,2,\ldots ,m\};~a\ne b)$ exist, where $\Phi$ is an empty set. The system characteristic moment of the correct branch on all *m* characteristic points is $\sum _{i=1}^m N_{i}=n-1$, and the system characteristic moment of any interference branch $j\in {\mathbf {S}}_x(x\in \{1,2,\ldots ,m\})$ on all *m* characteristic points is $\sum _{i=1,i \ne x}^m N_{i}+(n-1)$. Since $n-1<\sum _{i=1,i \ne x}^m N_{i}+(n-1)$, the system characteristic moment of the correct branch is unique and the smallest.

We can further assert that $m < n$. In addition to the correct branch, the designer of the decision event also needs to design the number of $(n-1)$ interference branches. As each interference branch can only be changed according to one feature point of the correct branch, if $m = n$, *m* feature points are used to distinguish $n-1$ interference branches. According to the Pigeonhole principle, there must be two feature points that are distinguishable on $n-1$ interference branches, so there are indeed redundant feature points.

## Conclusion

When facing a multiple-choice question with completely unfamiliar background knowledge or internal mechanism, we find that there is a psychological tendency to observe and select branch features and find the most common features as the decision outcome, and the decision outcome of this method is often the correct answer. In this paper, firstly, this type of phenomenon is normalized and quantified, which is named the zero-knowledge decision. Further, it is deeply discussed from four aspects, that is, (1) universality of the cases with the ZKDM method; (2) extensity of the use of the ZKDM method in situations where it is potentially adoptable? (3) Mechanism behind the ZKDM method; (4) correctness of the ZKDM method.

Through data investigation, it is observed that the cases selected correctly by the ZKDM method have a certain universality. This is a practical but often overlooked feature for the designer of decision events, which allows decision-makers to arrive at the correct option using ZKDM. However, there are still some limitations in the collection of cases with the zero-knowledge decision. It is also necessary to make statistics of large sample data, analyze the proportion of cases with the zero-knowledge decision in daily decision events and the accuracy of decisions.

It is proposed that the reason for the existence and high accuracy of zero-knowledge decision is that the designers of the decision events have the design habit of “making slight changes of the correct option to design interference options”. Therefore, the zero-knowledge decision depends on intuitive psychological tendency from the appearance, but in fact, there is a rational logical mechanism behind it. In the zero-knowledge decision event, the cause (all selection branches) of the decision event is the effect (the correct branch) of the effect (the changes corresponding to the correct branch) from the perspective of the event designer. From the point of view of the event decision-maker, the effect of the decision event (the correct branch) is the cause (all selection branches) of the cause (arising from the correct branch). Through a questionnaire survey of 279 event designers, the results show that more than half (63.80%) adopt the idea of “the cause is the effect of effect” in event design, indicating a high degree of universality. According to the questionnaire survey of 465 event decision-makers, 89 candidates, accounting for 19.14% in the total number, are able to use the ZKDM method to make choices. This result indicates that one in five people in the crowd has the thinking of the ZKDM. Simultaneously, the results of the questionnaire also present that there are a small number of decision-makers (4 candidates) who not only “know what it is” but also “know why it is”. This result may deduce that they make decisions from the thinking height of “the effect is the cause of cause”. For example, there are such descriptions in the questionnaire: “From a psychological point of view, options 1, 2, and 4 of the WeChat logo are all obtained by a small modification of option 3”, “There is only one difference between the interference options and the correct option”, “Option 3 has the characteristics of the other three pictures according to the rule of the question”. In addition, in the Questionnaire 2, decision-makers choosing the correct option also show a decision-making method based on memory, preference and inspire. Further, keywords like *impression, familiarity, overall similarity, memory, experience, intuition, feeling, unnaturalness, incongruity, symmetry, visual habit, beauty, pleasing to the eye, comfort, substitution sense, hierarchy sense, rationality, design principle of logo, the limit of space and time of communication, privacy, distance sense, association* appear in the text description of ideas and practices for some respondents. For these reasons, ZKDM is an important and emerging field of research in determining human behaviours in decision making.

Here, it should be noted that the current survey results of these questionnaires are still based on small samples, and the survey objects are relatively single (college students and college teachers), which does not have universality. The experience, intelligence and knowledge level of decision makers may affect the formation of zero knowledge decision-making psychology (i.e. leaning towards the most common features). Decision makers are based on their own cognition of the world, and are inspired by the transfer of underlying logic and knowledge (such as simplicity, similarity and optimality), then the zero- knowledge decision-making psychology is formed imperceptibly.

The mechanism behind the existence of zero-knowledge decision events is that the designer of decision events adopts the design idea of “changing the correct answer and deriving other selection branches”. Therefore, the correctness of zero-knowledge decision will also depend on the designer’s specific ”changing and deriving” scheme. For example, the designer only selects a feature point of the correct branch to change and generate interference branches, or select multiple feature points simultaneously to change to generate interference branches. There are many types of “changing and deriving “schemes. Aiming at one typical “changing and deriving “case, the correctness of the ZKDM method is proved in this paper. However, it should be noted that some types of “changing and deriving” schemes will lead to the situation that “the system characteristic moment of the correct branch is the smallest but not unique” and “the system characteristic moment of the correct branch is not the smallest”. In the former case, it is impossible to make the zero-knowledge decision because it is not unique. In the latter case, if we use the ZKDM method to select the branch with the smallest system characteristic moment, then the branch is not a correct branch, that is, the result of zero-knowledge decision is wrong.

## Supplementary Information

Supplementary Information.
