# Supplementary material for: Decision-making psychology and method under zero-knowledge context
Source: Sci Rep. 2022 Feb 24;12:3187. doi: 10.1038/s41598-022-06753-z (PMC8873423; doi:10.1038/s41598-022-06753-z)
Supplement: Supplementary file 1 — Supplementary Information. [file 41598_2022_6753_MOESM1_ESM.pdf]

Appendix: Examples

1. (National Paper I of Mathematics for Science in Chinese college entrance examination, 2017) Assume x, y, z as positive numbers, and $2^x = 3^y = 5^z$, then (D) holds.

A. $2x < 3y < 5z$ B. $5z < 2x < 3y$

C. $3y < 5z < 2x$ D. $3y < 2x < 5z$

First, we can find three feature points of the four options: (i) the minimum value; (ii) the median value; and (iii) the maximum value. Then, for each feature point, a feature set can be built to describe the features of the four options. The feature set based on the first feature point is {2x, 5z, 3y, 3y}. The feature set based on the second feature point is {3y, 2x, 5z, 2x}. The feature set based on the third feature point is {5z, 3y, 2x, 5z}. Second, the characteristic moments of the four selection branches based on the first feature point are (3,3,2,2). The characteristic moments of the four selection branches based on the second feature point are (3,2,3,2); The characteristic moments of the four selection branches based on the third feature point are (2,3,3,2). By summing up the characteristic moments of the above three feature points, the characteristic moments of the system based on the four selection branches of all feature points can be obtained as (8, 8, 8, 6). Finally, the system characteristic moment of option D is the smallest. So the decision-maker should choose option D and the correct answer is indeed option D.

2. (National Paper I of mathematics of liberal arts in Chinese college entrance examination, 2018) Assume the function $(x) = \{\begin{matrix} 2^{-x}, x \le 0 \\ 1, x > 0 \end{matrix}$. Then, the scope of x should be (D) when satisfying $f(x+1) < f(2x)$.

A. (-∞,-1] B. (0, +∞)

C. (-1,0) D.(-∞,0)

First, we can find two feature points of the four options: (i) the value of the left endpoint of the interval; (ii) the value of the right endpoint of the interval. Then, for each feature point, a feature set can be built to describe the features of the four options. The feature set based on the first feature point is {-∞, 0, -1, -∞}. The feature set based on the second feature point is {-1, +∞, 0, 0}. Second, the characteristic moments of the four selection branches based on the first feature point are (2,3,3,2). The characteristic moments of the four selection branches based on the second feature point are (3,3,2,2). By summing up the characteristic moments of the above two feature points, the characteristic moments of the system based on the four selection branches of all the two feature points can be obtained as (5,6,5,4). Finally, the system characteristic moment of option D is the smallest. So the decision-maker should choose option D and the correct answer is indeed option D.

3. (National Paper I of Mathematics for Science in Chinese college entrance examination, 2018) Given that the edge length of a cube is 1, and the angle formed by the straight line of each edge and a plane α is equal, then the maximum cross-sectional area of α across the cube is (A).

A. $\frac{3\sqrt{3}}{4}$ B. $\frac{2\sqrt{3}}{3}$ C. $\frac{3\sqrt{2}}{4}$ D. $\frac{\sqrt{3}}{2}$

First, we can find two feature points of the four options: (i) the denominator; and (ii) the numerator. Then, for each feature point, a feature set can be built to describe the features of the four options. The feature set based on the first feature point is {3/4,2/3,3/4,1/2}. The feature set based on the second feature point is $\{\sqrt{3}, \sqrt{3}, \sqrt{2}, \sqrt{3}\}$. Next, the characteristic moments of the four selection branches based on the first feature point are (2,3,2,3). The characteristic moments of the four selection branches based on the second

feature point are (1,1,3,1). By summing up the characteristic moments of the above two feature points, the characteristic moments of the system based on the four selection branches of all the two feature points can be obtained as (3,4,5,4). Finally, the system characteristic moment of option A is the smallest. So the decision-maker should choose option A and the correct answer is indeed option A.

4. (National Paper III of Mathematics for Science in Chinese college entrance examination, 2017) Given that the function $f(x) = x^2 - 2x + a(e^{x-1} + e^{-x+1})$ has a unique zero point, then a = (C)

A. $-\frac{1}{2}$ B. $\frac{1}{3}$ C. $\frac{1}{2}$ D. 1

First, we can find two feature points for the four options: (i) the symbol; and (ii) the number. Then, for each feature point, a feature set can be built to describe the features of the four options. The feature set based on the first feature point is {-, +, +, +}. The feature set based on the second feature point is {1/2,1/3,1/2,1}. Next, the characteristic moments of the four selection branches based on the first feature point are (3,1,1,1). The characteristic moments of the four selection branches based on the second feature point are (2,3,2,3). By summing up the characteristic moments of the above two feature points, the characteristic moments of the system based on the four selection branches of all the two feature points can be obtained as (5,4,3,4). Finally, the system characteristic moment of option C is the smallest. So the decision-maker should choose option C and the correct answer is indeed option C.

5. If the function $f(x) = a^x - x^a$ $(a > 0, a \neq 1, x > 0)$ has only one zero point, then the value range of the real number *a* is (B).

A. (0,1)∪(1,e] B.(0,1) ∪{e}

C.(0,e/3] ∪{e} D.(1,e] ∪{e^2}

First, we can find two feature points of the four options : (i) the first interval; and (ii) the second interval. Then, for each feature point, a feature set can be built to describe the features of the four options. The feature set based on the first feature point is {(0, 1), (0, 1), (0,e/3], (1,e]}. The feature set based on the second feature point is {(1,e],{e},{e},{e^2}}. Next, the characteristic moments of the four selection branches based on the first feature point are (2,2,3,3). The characteristic moments of the four selection branches based on the second feature point are (3,2,2,3). By summing up the characteristic moments of the above two feature points, the characteristic moments of the system based on the four selection branches of all the two feature points can be obtained as (5,4,5,6). Finally, the system characteristic moment of option B is the smallest. So the decision-maker should choose option B and the correct answer is indeed option B.

6. As is shown in the figure, the car moves in a straight line along the horizontal ground. The angle between the suspension line of the ball suspended on the roof of the car and the vertical direction is denoted as θ. An object A with mass *m* is placed on the floor of the car, and it is relatively stationary with the car. The magnitude and direction of the friction force on object A is (B).

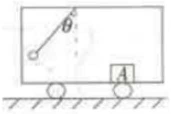

A. *mgsinθ*, towards the right

B. *mgtanθ*, towards the right

C. *mgcosθ*, towards the right

D. *mgtanθ*, towards the left

First, we can find two feature points for the four options: (i) the magnitude of the friction force; and (ii) the direction of the friction force. Then, for each feature point, a feature set can be built to describe the features

of the four options. The feature set based on the first feature point is {mgsinθ, mgtanθ, mgcosθ, mgtanθ}. The feature set based on the second feature point is {right, right, right, left}. Next, the characteristic moments of the four selection branches based on the first feature point are (3,2,3,2). The characteristic moments of the four selection branches based on the second feature point are (1,1,1,3). By summing up the characteristic moments of the above two feature points, the characteristic moments of the system based on the four selection branches of all the two feature points can be obtained as (4,3,4,5). Finally, the system characteristic moment of option B is the smallest. So the decision-maker should choose option B and the correct answer is indeed option B.

7. David discovered "laughing gas" (N_2O) while studying the effects of gas on the human body. Then, the combined valence of Nitrogen in N_2O is (C).

A.0

B. -1

C. +1

D. +2

First, we can find two feature points for the four options: (i) the symbol; and (ii) the number. Then, for each feature point, a feature set can be built to describe the features of the four options. The feature set based on the first feature point is {none, -, +, +}. The feature set based on the second feature point is {0,1,1,2}. Next, the characteristic moments of the four selection branches based on the first feature point are (3,3,2,2). The characteristic moments of the four selection branches based on the second feature point are (3,2,2,3). By summing up the characteristic moments of the above two feature points, the characteristic moments of the system based on the four selection branches of all the two feature points can be obtained as (6,5,4,5). Finally, the system characteristic moment of option C is the smallest. So the decision-maker should choose option C and the correct answer is indeed option C.

8. The way that the blood enters the kidneys and forms urine is (C)

A. Plasma → renal capsule → glomerulus → renal tubule → urine

B. Plasma → renal tubule → renal capsule → glomerulus → urine

C. Plasma → glomerulus → renal capsule → renal tubular → urine

D. Plasma → glomerulus → renal tubule → renal capsule → urine

First, we can find three feature points for the four options: (ii) the second part of the pathway; (ii) the third part of the pathway; and (iii) the fourth part of the pathway. Then, for each feature point, a feature set can be built to describe the features of the four options. The feature set based on the first feature point is {renal capsule, renal tubule, glomerulus, glomerulus}. The feature set based on the second feature point is {glomerulus, renal capsule, renal capsule, renal tubule}. The feature set based on the third feature point is {renal tubule, glomerulus, renal tubule, renal capsule}. The characteristic moments of the four selection branches based on the first feature point are (3,3,2,2). The characteristic moments of the four selection branches based on the second feature point are (3,2, 2,3). The characteristic moments of the four selection branches based on the third feature point are (2,3,2,3). By summing up the characteristic moments of the above three feature points, the characteristic moments of the system based on the four selection branches of all three feature points can be obtained as (8,8,6,8). Finally, the system characteristic moment of option C is the smallest. So the decision-maker should choose option C and the correct answer is

indeed option C.

9. The Spring Festival Gala of the Year of the Sheep, a Chinese zodiac symbol in traditional Chinese culture, was staged grandly in the studio hall of CCTV at 8 pm on February 18, 2015. The wonderful program attracted the attention of Chinese people all over the world. Accordingly, the local time (zone time) when the Chinese and overseas Chinese in San Francisco (37°N 122°W), USA began to watch the live broadcast of the Spring Festival Gala was (A)

A. 4 am on February 18

B. 4 am on February 19

C. 4 am on February 17

D. 8 pm on February 18

First, we can find two feature points for the four options: (i) date; and (ii) time. Then, for each feature point, a feature set can be built to describe the features of the four options. The feature set based on the first feature point is {February 18, February 19, February 17, February 18}. The feature set based on the second feature point is {4 am,4 am, 4 am, 8pm }. Next, the characteristic moments of the four selection branches based on the first feature point are (2,3,3,2). The characteristic moments of the four selection branches based on the second feature point are (1,1,1,3). By summing up the characteristic moments of the above two feature points, the characteristic moments of the system based on the four selection branches of all the two feature points can be obtained as (3,4,4,5). Finally, the system characteristic moment of option A is the smallest. So the decision-maker should choose option A and the correct answer is indeed option A.

10. In modern Chinese history, the country that occupied the most territory of China, and the unequal treaties that opened the most treaty ports and paid the most reparations were respectively (B)

A. Britain, Treaty of Nanking, Treaty of Sinchou

B. Tsarist Russia, Treaty of Tientsin, Treaty of Sinchou

C. Tsarist Russia, Treaty of Tientsin, Treaty of Shimonoseki

D. Britain, Treaty of Peking, Treaty of Sinchou

First, we can find three features of the four options: (i) the country that occupied the most territory of China; (ii) the treaty that opened the most trading ports; and (iii) the treaty with the most compensation. Then, for each feature point, a feature set can be built to describe the features of the four options. The feature set based on the first feature point is {Britain, Tsarist Russia, Tsarist Russia, Britain}. The feature set based on the second feature point is {Treaty of Nanking, Treaty of Tientsin, Treaty of Tientsin, Treaty of Peking}. The feature set based on the third feature point is {Treaty of Sinchou, Treaty of Sinchou, Treaty of Shimonoseki, Treaty of Sinchou}. Next, the characteristic moments of the four selection branches based on the first feature point are (2,2,2,2). The characteristic moments of the four selection branches based on the second feature point are (3,2,2,3). The characteristic moments of the four selection branches based on the third feature point are (1,1,3,1). By summing up the characteristic moments of the above three feature points, the characteristic moments of the system based on the four selection branches of all three feature points can be obtained as (6,5,7,6). Finally, the system characteristic moment of option B is the smallest. So the decision-maker should choose option B and the correct answer is indeed option B.

11. In terms of ideology and culture, the founding of Marxism mainly critically inherited and absorbed the reasonable ideas of

what theories? (C)

A. British classical philosophy; British classical political economics; Utopian socialism in France and Germany

B. German classical philosophy; French classical political economics; Utopian socialism in France and Britain

C. German classical philosophy; British classical political economics; Utopian socialism in France and Britain

D. French classical philosophy; German classical political economics; Utopian socialism in France and Britain

First, we can find three characteristic points for the four options: (i) the first thought; (2) the second thought; (3) The third thought. Then, for each feature point, a feature set can be built to describe the features of the four options. The feature set based on the first feature point is { British classical philosophy, German classical philosophy, German classical philosophy, French classical philosophy}. The feature set based on the second feature point is {British classical political economics, French classical political economics, British classical political economics, German classical political economics}. The feature set based on the third feature point is {Utopian socialism in France and Germany, Utopian socialism in France and Britain, Utopian socialism in France and Britain, Utopian socialism in France and Britain}. Next, the characteristic moments of the four selection branches based on the first feature point are (3,2,2,3). The characteristic moments of the four selection branches based on the second feature point are (2,3,2,3). The characteristic moments of the four selection branches based on the third feature point are (3,1,1,1). By summing up the characteristic moments of the above three feature points, the characteristic moments of the system based on the four selection branches of all three feature points can be obtained as (8,6,5,7). Finally, the system characteristic moment of option C is So the decision-maker should choose option C and the correct answer is indeed option C.

12. It's seven thirty, I __B__ go to school.

A. have got

B. have got to

C. got to

D. had got to

First, we can find two feature points for the four options: (i) tense; and (ii) collocation. Then, for each feature point, a feature set was built to describe the features of the four options. The feature set based on the first feature point is {present perfect tense, present perfect tense, past tense, past perfect tense}. The feature set based on the second feature point is {got, got to, got to, got to}. Next, the characteristic moments of the four selection branches based on the first feature point are (2,2,3,3). The characteristic moments of the four selection branches based on the second feature point are (3,1,1,1). By summing up the characteristic moments of the above two feature points, the characteristic moments of the system based on the four selection branches of all the two feature points can be obtained as (5,3,4,4). Finally, the system characteristic moment of option B is the smallest. So the decision-maker should choose option B and the correct answer is indeed option B.

13. She would rather have a room ____B__ than sleep with her sister.

A. on her own B. of her own

C. of hers D. for her

First, we can find two feature points for the four options: (i) preposition; (2) person. Then, for each feature point, a feature set can be built to describe the features of the four options. The feature set based on the first feature point is {on, of, of, for}. The feature set based on the second feature point is {her

own, her own, hers, her}. Next, the characteristic moments of the four selection branches based on the first feature point are (3,2,2,3). The characteristic moments of the four selection branches based on the second feature point are (2,2,3,3). By summing up the characteristic moments of the above two feature points, the characteristic moments of the system based on the four selection branches of all the two feature points can be obtained as (5,4,5,6). Finally, the system characteristic moment of option B is the smallest. So the decision-maker should choose option B and the correct answer is indeed option B.

14. I'll never forget the days ___B___ I lived in the country with the farmers, ___ has a great effect on my life.

A. that; which B. when; which

C. which; that D. when; who

First, we can find two feature points for the four options: (i) conjunction of the first subordinate clause; and (ii) conjunction of the second clause. Then, for each feature point, a feature set can be built to describe the features of the four options. The feature set based on the first feature point is {that, when, which, when}. The feature set based on the second feature point is {which, which, that, who}. The characteristic moments of the four selection branches based on the first feature point are (3,2,3,2). The characteristic moments of the four selection branches based on the second feature point are (2,2,3,3). By summing up the characteristic moments of the above two feature points, the characteristic moments of the system based on the four selection branches of all the two feature points can be obtained as (5,4,6,5). Finally, the system characteristic moment of option B is the smallest. So the decision-maker should choose option B and the correct answer is indeed option B.
